# Treatment of Unfavorable Intrabony Defects with Autogenous Bone Graft in Combination with Leukocyte- and Platelet-Rich Fibrin or Collagen Membranes: A Non-Inferiority Study

**DOI:** 10.3390/medicina60071091

**Published:** 2024-07-03

**Authors:** Giuseppe Balice, Michele Paolantonio, Paolo De Ninis, Imena Rexhepi, Matteo Serroni, Alessio Frisone, Luigi Romano, Bruna Sinjari, Giovanna Murmura, Beatrice Femminella

**Affiliations:** 1 Department of Innovative Technologies in Medicine and Dentistry, “G. D’Annunzio” University, 66100 Chieti-Pescara, Italy; giuseppe.balice@phd.unich.it (G.B.); michele.paolantonio@unich.it (M.P.); imena.rexhepi@unich.it (I.R.); matteo.serroni@phd.unich.it (M.S.); ales.frisone@gmail.com (A.F.); luigi.romano@unich.it (L.R.); b.sinjari@unich.it (B.S.); giovanna.murmura@unich.it (G.M.); 2 “Luisa D’Annunzio” Institute for High Culture, 65123 Pescara, Italy

**Keywords:** bone transplantation, fibrin, randomized controlled trial, regeneration, wound healing

## Abstract

*Background and Objectives*: Unfavorable intrabony defects (IBDs) are associated with the progression of periodontal disease and tooth loss. Growing scientific evidence has demonstrated the effectiveness of platelet concentrations in periodontal treatment. The aim of our study was to demonstrate the non-inferiority of an autogenous bone graft (ABG) associated with leukocyte- and platelet-rich fibrin (L-PRF) compared to ABG + Collagen Membrane in the treatment of IBDs. *Material and Methods*: Sixty-four patients with at least one IBD were randomly assigned to two groups: ABG+L-PRF and CM+ABG. Clinical and radiographic evaluations were performed at baseline and 12-month follow-up. Clinical attachment level (CAL), gingival recession (GR), probing pocket depth (PPD), and radiograph defect bone level (DBL) were compared between the two treatments. To evaluate the effectiveness of ABG+L-PRF, a non-inferiority margin of =1 mm (−1 mm for GR) was chosen; a second non-inferiority margin of =0.5 mm (−0.5 mm for GR) was set for clinical relevance. *Results*: At 12-month follow up, both treatments showed clinical and radiographic improvements. The 90% confidence intervals of the CM+ABG–L-PRF+ABG mean difference for CAL gain (−0.0564 mm [−0.316 to 0.203]), DBL gain (−0.433 mm [−0.721 to −0.145]), and PPD reduction (0.232 mm [0.015 to 0.449]) were below the 0.5 mm non-inferiority margin; the GR increase (0.255 mm [0.0645 to 0.445]) stayed above the −0.5 mm. *Conclusions*: the L-PRF+ABG treatment of unfavorable IBDs is non-inferior with respect to the CM+ABG therapy for CAL gain, but with a lower GR, a slightly higher PPD, and DBL gain.

## 1. Introduction

Periodontitis is a multifactorial inflammatory disease that leads to the loss of periodontal bone support. Deep infrabony defects (IBDs) are a common consequence of periodontal damage and are associated with the progression of periodontitis and tooth loss. This condition results not only in insufficient masticatory function but also in impaired speech and aesthetics, negatively impacting patients’ social relationships [1,2,3].

According to the classification by Goldman & Cohen [4], intrabony defects are categorized as follows: (i) one-wall intrabony defects: defects bounded by one osseous wall and the tooth surface; (ii) two-wall intrabony defects: defects bounded by two osseous walls and the tooth surface; (iii) three-wall intrabony defects: defects bounded by three osseous walls and the tooth surface.

After patient supragingival dental biofilm control (step 1 of periodontal therapy) [5], scaling and root planing (SRP) is the second step in the active treatment of periodontitis [6]. It aims to eliminate etiological factors, such as subgingival plaque and calculus. However, persistent pathological pockets are often associated with an infrabony pattern of bone resorption, necessitating surgical periodontal treatment [7].

Open flap debridement (OFD) is the most common periodontal surgical treatment for removing subgingival etiological factors, providing better accessibility to the deep component of the infrabony defect and resulting in a reduction of pocket probing depth (PPD) [7]. Histological studies have shown that healing achieved via OFD involves the formation of a long junctional epithelium on the previously treated root surface [8,9], rather than the regeneration of periodontal tissue, including new attachment, cementum, and periodontal ligament [10].

In contrast, regenerative surgical procedures aim to achieve the restitutio ad integrum of periodontal damaged tissues by recreating lost attachment tissue. Guided tissue regeneration (GTR) and Enamel Matrix Derivative (EMD) are two effective techniques for periodontal regeneration [11,12,13,14,15]. GTR, using a mechanical barrier, aims to block the apical migration of epithelial cells and stabilize the blood clot [16,17]. Resorbable membranes, such as collagen membranes (CMs), are widely used in clinical practice due to their ability to retain the benefits of non-absorbable membranes while overcoming their drawbacks [16].

An alternative to GTR is growth-factor-induced regeneration. Recent evidence has demonstrated the effectiveness of biological mediators, such as platelet concentrates, in stimulating certain cell anabolic activities during periodontal reconstructive therapy. Leukocyte- and platelet-rich fibrin (L-PRF) is a second-generation autologous platelet concentrate obtained from a simple venous blood sample. It is inexpensive, easy to prepare, and painless for the patient, with excellent periodontal-regeneration-promoting properties [18,19]. The membranes obtained by squeezing the fibrin clot can be used to cover bone defects or mixed with a filling material to enhance its regenerative characteristics.

However, both collagen and L-PRF membranes are not rigid, and thus lack space-making properties. Therefore, different grafts can be added to the membranes when the bony defect is not contained to enhance blood clot stability [20]. Among the biomaterials for the treatment of unfavorable infrabony defects, autologous bone graft (ABG) still represents the gold standard [21]. Despite its limitations, which are associated with a limited availability and post-operative complications such as swelling and pain for the patient, ABG combines the three key aspects of tissue engineering: scaffolds, stem cells, and growth factors, favoring osteoinductive, osteoconductive, and osteogenic conditions [22,23]. The effectiveness of using ABG associated with CM or L-PRF to produce periodontal regeneration in deep IBDs is well-documented [24,25]. Given these recent findings in the literature, the hypothesis of this study was to demonstrate the non-inferior clinical attachment level (CAL) gain achieved with ABG and L-PRF compared to ABG and CM, assessed at 1 year after surgery.

## 2. Materials and Methods

### 2.1. Experimental Design

This study was designed as a masked, parallel two-arm randomized non-inferiority clinical trial aimed to evaluate the 12-month clinical and radiographic outcomes for the treatment of unfavorable IBDs using two methods: L-PRF+ABG (test treatment) and CM+ABG (active comparator treatment). Since a previous study [26] demonstrated the non-inferiority of L-PRF against CM when both were associated with Inorganic Bovine Bone (IBB), in this study, we aimed to evaluate whether the same result was obtained when we used ABG, which represents the gold standard of filling materials for bone regeneration [21]. Historically, OFD served as placebo against which to demonstrate effectiveness. The primary outcome considered was CAL gain, and the secondary outcomes evaluated were PPD, Gingival Recession (GR), and Defect Bone Level (DBL). The outcomes were relevant to both a per-protocol and an intention-to-treat population.

The null hypothesis was:

*H*_0_: *µ*_*CM*+ABG_ − *µ*_*L* − *PRF*+ABG_ ≥ Δ_*NI*_ (1)

The effect of the active comparator was larger than that of the new treatment by at least one Δ_*NI*_.

The alternative hypothesis was as follows:

*H*_1_: *µ*_*CM*+ABG_ − *µ*_*L* − *PRF*+ABG_ < Δ_*NI*_ (2)

### 2.2. Non-Inferiority Margin

**Figure 1:**
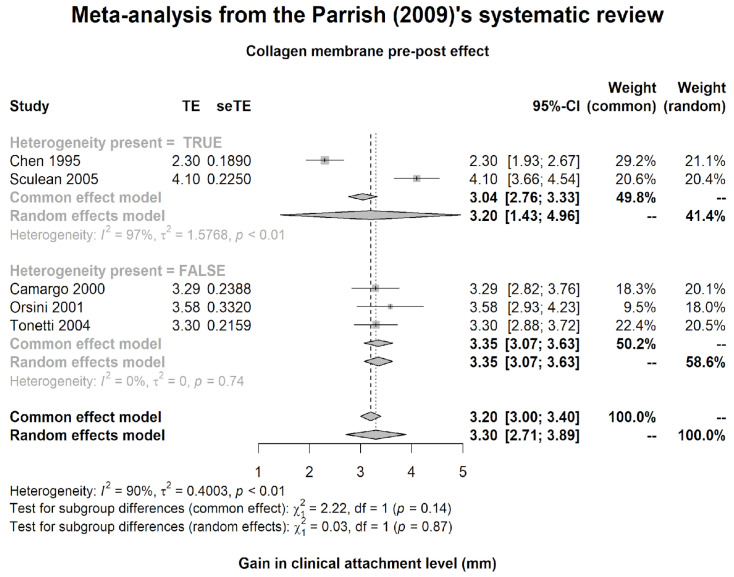
From Rexhepi [26], data by Parrish (2009) [29], excluding Vouros for SD unavailability, with comparison of two subgroups with maximal and minimal heterogeneity.

Since it has been demonstrated in the literature that the regenerative efficacy induced by IBB is comparable to ABG [27,28], an estimate of CAL gain provided from the CM+IBB treatment of

(3) $$M_{CM+IBB}=3.30\pm SD=1.11\;95\%\;\mathrm{CI}\;(2.71\;\mathrm{to}\;3.89)$$

was obtained by re-analyzing a random effects meta-analysis on data by Parrish et al. [29], available in Rexhepi et al. [26], and shown in Figure 1.

For the placebo arm OFD surgical treatment, the mean estimate from the literature [30] was *M*_*OFD*_ = 2.48 mm.

The differential effect of adding GTR was 0.82 mm, rounded to 1 mm.

According to the so-called fixed margin approach [31], a margin ∆M_1_ = 1 mm was therefore defined to ensure the new treatment efficacy versus OFD (30% of the overall effect). To maintain an additional 50% of the comparator effect (15% of the total), a second, narrower margin, ∆M_2_ = 0.5 mm (degree of inferiority), was specified.

For DBL and PPD, equal non-inferiority margins were set, while for GR, where lower values are better, the margins were −1 mm and −0.5 mm.

### 2.3. Sample Size

Sixteen patients per group are needed to reject the H_0_ with respect to the bigger margin ΔM_1_ in a one-sided test with *α* = 0.05, 1−β=0.80, and an SD = 1.11 mm [29].

To set a halved margin ΔM_2_ = 0.5 mm, the sample size needs to be increased four times. Instead, when the collected data meet the assumptions of an ANCOVA with the baseline values as covariate, these values account for half the response variance thanks to their theoretical correlation with the gain-scores *ρ* = 0.707 [32]. Consequently, the required sample size only doubles [33].

Sixty-two patients are required to be 80% certain that the upper limit of a one-sided 95% confidence interval (CI) of their difference will be below the non-inferiority margin of 0.5 mm (and >95% sure that it will be below the non-inferiority margin of 1 mm) if the standard treatment is truly not clinically superior. We enrolled 64 patients.

### 2.4. Study Population

Sixty-four patients were enrolled in this study; they were selected from a population of 180 patients affected by stage III-IV periodontitis [34] who presented at the Unit of Periodontology of the G. D’Annunzio” University between June 2018 and June 2022.

### 2.5. Patient’s Inclusion and Exclusion Criteria

The inclusion criteria were as follows: (1) age ≥ 18 years; (2) good systemic condition; (3) full-mouth plaque score (FMPS) and full-mouth bleeding score (FMBS) < 20% at surgery; (4) >20 teeth without dental mobility; (5) non-smoker or former smoker for ≥10 years; (6) at least one site that radiographically showed vertical bone loss (level of the alveolar crest-bottom defect distance) ≥ 4 mm and at PD ≥ 5 mm 12 weeks after non-surgical treatment; (7) no periapical lesion at the experimental site.

The exclusion criteria were: (1) drugs that may affect periodontal status in the previous 6 months; (2) periodontal treatment for at least 2 years; (3) pregnancy/lactation.

The IBDs considered were primarily 1-wall defects, combined 1–2 wall defects, and 2-wall defects/craters, circumferential defects (including at least three surfaces), or teeth with a large defect angle (≥36°) [35], which were taken into consideration in this study. Surgical intervention was required to confirm the anatomy. Each patient participated in the study with a single experimental site. Informed consent was obtained from all patients participating in the study. All patients received Scaling and Root Planing (SRP) using ultrasonic instruments with periodontal tips and Mini-Five Gracey curettes; furthermore, customized oral hygiene instructions were given to each patient.

The research was conducted in accordance with the 1975 Helsinki Declaration, as amended in 2013, and was authorized by G. D’Annunzio University’s ethical committee (n°025062018-07/05/2018). The study was conducted from June 2018 to July 2023. This clinical trial was registered at ClinicalTrial.gov as NCT04043754.

#### Randomization and Blinding Protocol

A blood sample, necessary for the L-PRF+ABG treatment, was taken from all patients. Patients and examiners were masked to group membership; clinical and radiographic examiners were masked to each other; the study analyst was masked to group membership.

The trial director oversaw the random assignment of patients to treatment groups after enrollment and was not involved in the clinical interventions or study measurements. A computer-generated table was used to make the random assignment, which was known only to the trial director. An opaque envelope, concealing group allocation, was opened just before surgery, so the surgeon was masked to group membership until that moment and was not involved in any way in the collection of clinical data. The data analyst did not know the group to which each analyzed patient belonged. After receiving the data for groups A and B, the analyst produced two 90% confidence intervals (CIs) for the differences (A minus B and vice versa). The blinding was not removed until the study was finished, and the correct difference was maintained.

### 2.6. Clinical Measurements

The clinical measurements performed were CAL, PPD, GR, FMBS, and FMPS, and they were evaluated at 3 months from SRP and 1 year after surgery. The measurements were recorded by the same expert operator GB using a UNCP-15-mm periodontal probe.

### 2.7. Radiographic Measurement

A 70-kV intraoral X-ray device with a digital sensor and an exposure period of 0.12 s was used to acquire periapical radiographs. Intraoral standardized radiographs were obtained before and 12 months after SRP using the long-cone method and digital sensor holders specifically made for the selected experimental teeth. A thermoplastic occlusal bite block was used for reproducibility.

To calculate the distance between the alveolar crest level and the defect bottom, specific dental software was utilized.

### 2.8. Platelet-Rich Fibrin Preparation

For the platelet-concentrate-treated group, L-PRF was prepared following the protocol by Choukroun et al. [18,19]. A venous blood sample was taken from the antecubital vein immediately before surgery and placed into two 10 mL sterile tubes without anticoagulant. The blood sample was then centrifuged for 10 min at 3000 rpm using a centrifuge for medical use (Intra-Lock System Europe SpA, Salerno, Italy, called IntraSpinTM). To create L-PRF membranes, the fibrin clot was compressed into the L-PRF Box (XpressionTM Fabrication Kit, Intra-Lock System Europa SpA, Salerno, Italy). Therefore, one membrane was shredded and mixed with ABG; the other was used to cover the treated infrabony defect.

### 2.9. Surgical Procedure

**Figure 2:**
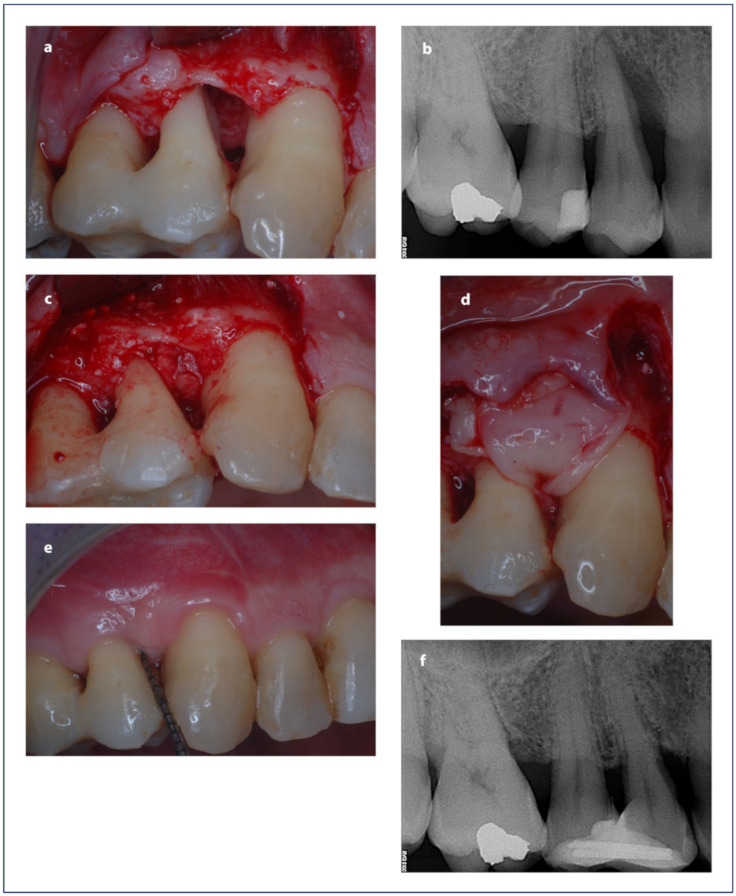
(**a**) The intrabony defect; (**b**) X-ray appearance of the defect; (**c**) the intrabony defect was filled with ABG mixed with L-PRF; (**d**) L-PRF membranes covers the graft; (**e**) clinical and (**f**) radiographic appearance of the treated site 1 year after surgery.

**Figure 3:**
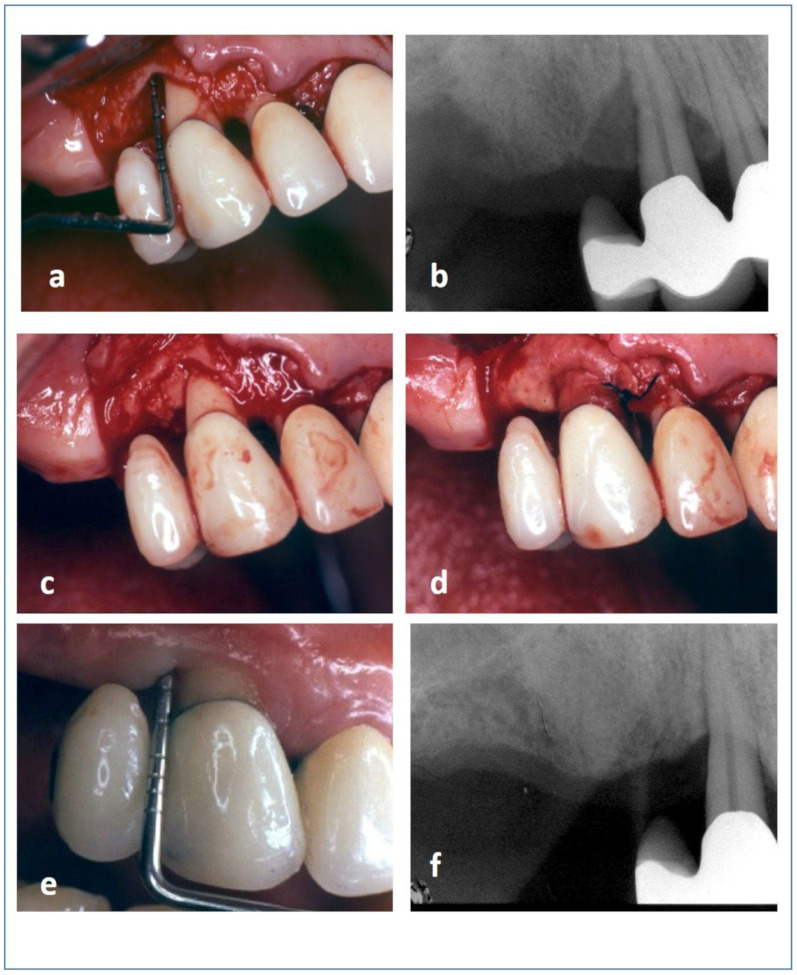
(**a**) The intrabony defect; (**b**) X-ray appearance of the defect; the intrabony defect was (**c**) filled with ABG and (**d**) covered with a CM; (**e**) clinical and (**f**) radiographic appearance of the treated site 1 year after surgery.

Each patient received surgery from the same expert surgeon (MP). The simplified papilla preservation flap procedure was performed to access the bone defect. At the vestibular side, intrasulcular incisions were made and continued obliquely over the papilla, intrasulcularly at the surrounding teeth. If required, a vertical releasing incision was made to complete the design of the flap. An intrasulcular palatal incision was performed at each tooth, confined to the mid-palatal aspect only. After flap elevation and bone defect degranulation, ABG was obtained from the surrounding intervention area using a safe-scraper and scraping the bone cortex, which is richer in bone morphogenetic proteins (BMPs) [36,37]. For the L-PRF test treatment, the IBD was filled with a graft made from a mixture of ABG and shredded L-PRF membrane; then, another L-PRF membrane was placed to cover the filled defect (Figure 2). Instead, in the active control group, after filling the defect with ABG only, a CM was used to cover the grafted defect (Figure 3).

In both procedures, after periosteal incisions, the tension-free flap was repositioned. A double suture was performed: a primary internal horizontal mattress suture was associated with a second interrupted suture to obtain primary intention healing. At the end of both surgical procedures, all patients received an injection of 4 mg of betamethasone to promote complication-free healing and reduce post-operative swelling.

### 2.10. Post-Operative Procedures

To prevent post-operative infections, all patients took 2 g/day of amoxicillin+clavulanic acid for six days. In addition, the patients were prescribed with 400 mg of ibuprofen twice a day for pain management as necessary, and with twice-a-day rinse with 0.20% chlorhexidine for three weeks. Sutures were removed at 14 days. Careful brushing with a soft toothbrush was permitted from two weeks following suture removal; interdental brushing was permitted after four weeks post-op; in the meantime, the patients utilized 1% chlorhexidine gel twice a day after oral hygiene procedures. The patients received motivational reinforcement and weekly supragingival professional hygiene for a period of six weeks. Up to the one-year examination, patients were kept clean by a professional cleaning every third month.

### 2.11. Statistical Analysis

**Figure 4:**
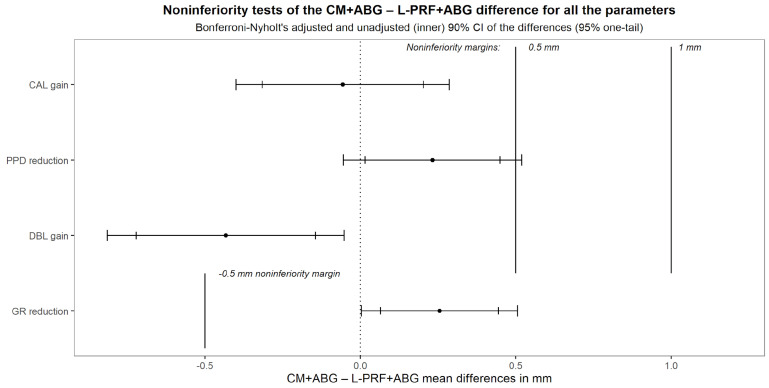
Simultaneous 90% CIs of adjusted differences between new treatment and active comparator. PPD, pocket probing depth; CAL, clinical attachment level; GR, gingival recession; DBL, defect bone level.

To find evidence of the new treatment non-inferiority, multiple univariate analyses of single outcomes were performed [38]. The difference between the treatment averages of the CAL gain was estimated using an ANCOVA adjusted for baseline with a second-degree polynomial term, and the 90% confidence interval was obtained. Non-inferiority was claimed if its upper bound was less than M_2_ = 0.5 mm. Secondary outcomes were analyzed similarly. Even though not appropriate for this study, which, not involving disjunction tests, cannot capitalize in chance [39], the following graph shows simultaneous CIs adjusted for the per-family error rate according to Bonferroni, adjusted in turn for the effective independent endpoint number according to Nyholt [40,41] (Figure 4).

The assumption of no covariate-by-treatment interaction, which was holding for the analyses of all the parameters, and the outcome–covariate correlation for the main outcome *ρ* = 0.81 allowed us to fit ANCOVAs and get sufficient power for M_2_ too. The quadratic term of the baseline covariate, unneeded for the DBL and GR regressions, was again necessary in the PPD model.

**Figure 5:**
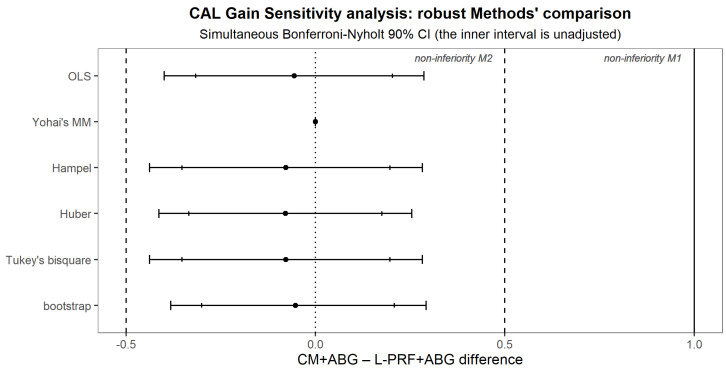
Sensitivity Analysis for the main outcome CAL.

For CAL gain, a sensitivity analysis comparing OLS to a set of robust estimation methods (bootstrap, Hampel, Huber, Tukey’s bisquare, and Yohai’s MM estimator), was performed to assess the robustness of the primary analysis findings (Figure 5).

## 3. Results

### 3.1. Study Population

**Figure 6:**
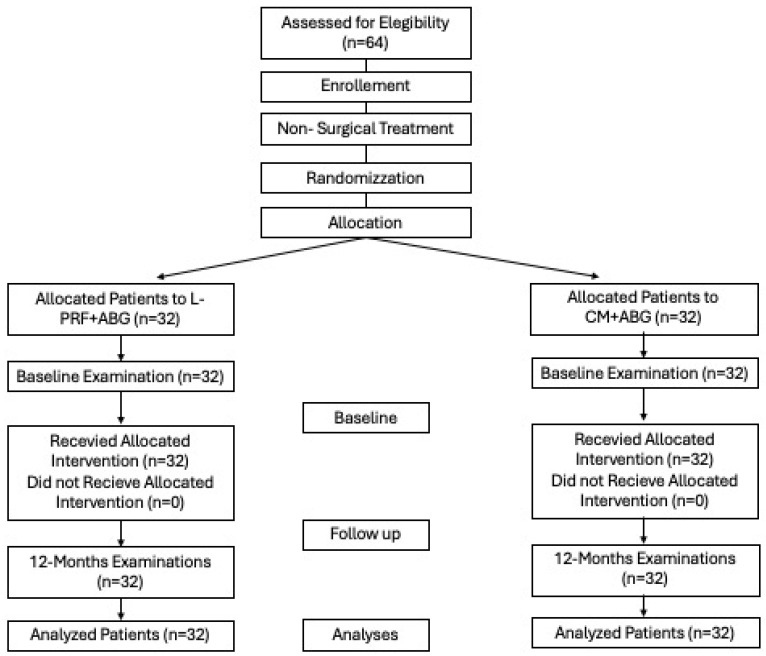
CONSORT FLOW diagram.

Figure 6 shows a CONSORT flow diagram, describing the research protocol. All 64 patients obtained the allocated treatment and completed the 12 months of follow-up without any drop-out and no post-operative complications. Therefore, the per-protocol and the intention-to-treat analyses coincide.

Observed means of all the parameters at baseline and follow-up along with their pre-post differences and the estimated means of the differences are shown in Table 1.

### 3.2. Clinical and Radiographic Outcomes

The anatomy of IBDs was confirmed intraoperatively.

No bleeding on probing occurred in any experimental sites for either group after 12 months.

No appreciable variations occurred within or between groups in FMPS and FMBS that stayed below 20% for all the duration of the trial. Table 2 displays the clinical and radiographic parameter values; both test and control defects showed a considerable improvement.

**Figure 7:**
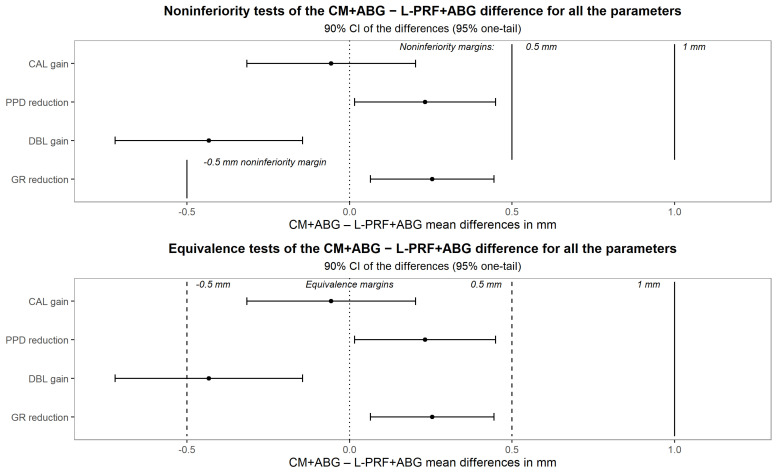
Upper: 90% CI for the difference CM+ABG–L-PRF+ABG between the treatment averages for all the parameters. Lower: equivalence tests of the CM+ABG–L-PRF+ABG differences for all the parameters.

The Figure 7 shows the 90% CI for the difference CM+ABG–L-PRF+ABG between the treatment averages for all the parameters.

The 95% upper bound (UB) for CAL gain was 0.203 mm, proving non-inferiority to the M_2_ margin. The 95% UB for the PPD difference was 0.449 mm, so non-inferior to M_2_. The 95% UB for DBL was −0.145 mm while the lower bound for GR was 0.0645 mm; both of them were not only non-inferior to their own M_2,_ but neither touched the zero line, involving the superiority of the L-PRF+ABG treatment. Interestingly, the CAL gain confidence interval was entirely contained even in the symmetrical negative margin of −0.5 mm, complying with the equivalence criterion too with respect to such not prespecified margin. The alternative hypothesis of this equivalence design appears fully appealing in that implies none of the treatments is inferior or superior to the other more than a 0.5 mm margin, therefore two conjunct one-tail significance tests (TOST) [42]. The same holds true for GR, whereby the new treatment resulted both equivalent and superior, and for PPD, where it resulted inferior, equivalent, and non-inferior, while for DBL it was superior, but failed the equivalence.

## 4. Discussion

### 4.1. Principal Findings

Our results suggest the effectiveness of both techniques in treating IBDs. The test treatment yielded non-inferior CAL gain improvements compared to the active comparator.

A crucial condition for the validity of a non-inferiority trial is the assumption that the active comparator indeed demonstrates its effect in the trial (assay sensitivity). Given the absence of the OFD arm, we needed to assess the comparator effect using historical data.

The effect observed in the CM+ABG group was 3.32 ± 0.134 mm (3.10 to 3.54), which is consistent with the estimate in the literature [29] of 3.30 mm (2.71 to 3.89), so the active comparator behaved according to expectation.

Since the reference population of our study, unfavorable IBDs, was not perfectly comparable with our literature reference, not specific for such defects, our results were probably particularly appreciable.

Regarding the risk of bias, the differences at baseline should have been suitably addressed by randomized allocation, even though the comparability of randomized groups in small samples cannot be taken as granted. In this case, the model included the baseline covariate; in the presence of randomized allocation, it provides a further guarantee.

### 4.2. Agreement and Disagreement with Previous Findings

To our knowledge, no study in the literature has compared guided tissue regeneration (GTR) with collagen membranes (CMs) and L-PRF in association with autogenous bone graft (ABG), considered the gold standard among filling biomaterials [21], in the treatment of infrabony defects. However, a non-inferiority study by Rexhepi et al. [26] is already present in the literature, which compared both techniques, but in association with IBB, demonstrating non-inferiority in clinical attachment level (CAL) gain of L-PRF compared to CM. A comparison between these two biomaterials was evaluated when covering the lateral access window after maxillary sinus lift, showing no difference between the two materials in bone formation [43,44]. Several recent studies in the literature [45,46] observed an additional effect of L-PRF + bone graft compared to L-PRF alone in CAL gain. This can be explained by the poor mechanical capacity of L-PRF in stabilizing the blood clot when it is not associated with a bone filler. Paolantonio in 2002 [47] showed the need to use a combined technique when the defect is not contained (≤2 walls) and the membrane risks collapsing into it, losing its space-making action. In these cases, the superiority of the combined technique compared to GTR alone in CAL gain is well reported. In a histological study, Sculean et al. [48] demonstrated how, in the case of uncontained IBDs, associating a bone graft with a membrane increased bone regeneration, compared to using the membrane alone. CM and L-PRF act in two different ways in determining periodontal regeneration. In the first case, the collagen membrane acts not so much as a mechanical obstacle to the migration of epithelial cells on the surface of the treated root, as was believed years ago [49], but rather as a primary stabilizer of the wound and the blood clot [50]. In contrast, L-PRF membranes release growth factors that induce and promote the regenerative mechanism. The presence of these soluble mediators such as vascular endothelial growth factor (VEGF), platelet-derived growth factor (PDGF), and fibroblast growth factor (FGF) [18,51] give the fibrin matrix of the L-PRF the ability to stimulate the angiogenic mechanism. Due to the rapid resorption of the L-PRF membrane, 1 or 2 weeks at most, the barrier effect is questionable; however, the ability of the PRF membrane to keep the BG in position within the IBDs and stabilize the blood clot, in the early stages of healing, is plausible.

### 4.3. Discussion of Secondary Outcomes

The secondary outcomes were evaluated in relation to the primary outcome CAL gain and therefore do not have full confirmatory value. However, the results obtained relating to DBL, GR, and PPD are noteworthy. L-PRF + ABG proved to be non-inferior, indeed superior, in terms of the increase in DBL (2.94 ± 0.76 mm), compared to the results obtained with the active comparator CM+ ABG (2.72 ± 0.85 mm). This is in accordance with the data from a previous study by Rexhepi et al. [26] (who used IBB) and can be justified by the high release of soluble growth factors present in L-PRF, which stimulate the regenerative mechanisms in a biologically active filler material such as ABG. A similar result was observed for GR, which was lower for the test treatment compared to the active comparator (0.66 ± 0.48 mm and 0.78 ± 0.71 mm, respectively), and this may be due to the trophic effects of L-PRF compared to CM; furthermore, the use of barrier membranes has been reported to be associated with increased GR [16,35]. The reduction in PPD was found to be greater in the active comparator group in comparison with the test group, probably as a consequence of the greater GR observed in the CM + ABG group. However, the clinical relevance of this result is questionable.

### 4.4. Clinical Implications

In the present study, we investigated the clinical effectiveness of two regenerative techniques in unfavorable infrabony defects (IBDs): 1–2 and circumferential defects, as well as IBDs with a large defect angle.

The healing potential of IBDs is strongly associated with the anatomy of the defect, the number of residual bony walls, and the width of the angle between the root and bone surface [52,53]. These aspects are essential for non-surgical therapy [6,52] and are also critical in periodontal regeneration, where the IBD’s ability to support and stabilize the blood clot is limited.

Several studies [12,35] have demonstrated that the number of bony walls of IBDs plays a marginal role in defect filling when treated by guided tissue regeneration (GTR). However, this applies when using a non-absorbable membrane such as ePTFE, which is more rigid than resorbable membranes like CM or L-PRF membrane. The latter tend to collapse within the defect, greatly reducing the tent effect, and therefore the space for regeneration [54]. Consequently, in the case of non-contained IBDs, the use of a graft is essential to support the resorbable membranes, ensuring the space maintenance necessary for tissue regeneration even after flap repositioning and suturing [55].

In a previous non-inferiority study, Rexhepi et al. [26] showed that L-PRF is non-inferior to CM in the combined regenerative therapy of unfavorable IBDs [56]. In the present study, we obtained the same result even when using a biomaterial considered as the gold standard for bone regeneration in the scientific literature.

This result does not surprise us since the scientific literature on filling biomaterials, used alone and without other regenerative aids, reports, on an exclusively clinical level, a substantial similarity of results in terms of clinical attachment level (CAL) gain among different bone fillers [57]. The present study, together with that of Rexhepi et al. [26], suggests that the same can happen when other regenerative aids are associated with different filling biomaterials. Naturally, this hypothesis needs to be confirmed in specifically designed clinical trials.

### 4.5. Limitations of the Study

The limitations of our study were that we didn’t employ stent-assisted probing, which would have certainly reduced the risk of errors caused by survey variability. Additionally, a split-mouth protocol was not utilized, which likely would have decreased the interindividual variability of the sample.

Furthermore, this study evaluated unfavorable bone defects, as defined in the literature [58], but did not select defects of similar and comparable architecture. Therefore, the IBDs treated in the two study groups were highly heterogeneous in their architecture. Since the sample size was very small, it was not feasible to use a randomized sample, which would have certainly reduced the risk of bias. Furthermore, the content of growth factors within the L-PRF membrane varies from patient to patient and is neither quantifiable nor standardizable [51].

Finally, being a clinical study, without histological control, nothing can be concluded about the actual quality of the tissues obtained through the regenerative procedures.

## 5. Conclusions

Our results confirmed what we had found in previous study published by us [26].

Both techniques were found to be effective in the treatment of IBDs, and L-PRF+ABG offers a CAL gain not inferior to the active comparator CM+ABG. It also has a much lower GR, which is a very useful aspect for clinicians working in anterior sextants. Nonetheless, a somewhat higher PD was noted. In conclusion, it can be agreed that the stabilization of the clot represents the main aspect of any form of healing, even when using ABG as the material to fill the unfavorable defect, considered today the gold standard of fillers materials.

## Figures and Tables

**Table 1:** Observed and Estimated Marginal Means in mm (Means ± SD) of the Clinical and Radiographic Parameter.

Parameter	Treatment	Baseline	12 Months	Baseline–12 Months	Baseline–12 Months
		Observed	Observed	Observed	Estimated Marginal
		Mean ± SD (95% CI)	Mean ± SD (95% CI)	Mean ± SD (95% CI)	Mean ± SD (95% CI)
**PPD**	L-PRF+ABG	7.47 ± 1.5 (6.93 to 8.01)	3.19 ± 0.78 (2.91 to 3.47)	4.28 ± 1.22 (3.84 to 4.72)	3.93 ± 0.65 (3.7 to 4.16)
	CM+ABG	7.44 ± 1.52 (6.89 to 7.99)	2.94 ± 0.62 (2.71 to 3.16)	4.5 ± 1.37 (4.01 to 4.99)	4.16 ± 0.66 (3.93 to 4.4)
	*diff*	*p = 0.93*	*p = 0.16*	*p = 0.50*	*p = 0.079*
**CAL**	L-PRF+ABG	8.28 ± 1.11 (7.88 to 8.68)	4.84 ± 0.85 (4.54 to 5.15)	3.44 ± 0.8 (3.15 to 3.73)	3.38 ± 0.67 (3.14 to 3.61)
	CM+ABG	8.66 ± 1.54 (8.1 to 9.21)	4.91 ± 0.73 (4.64 to 5.17)	3.75 ± 1.37 (3.26 to 4.24)	3.32 ± 0.76 (3.05 to 3.59)
	*diff*	*p = 0.27*	*p = 0.75*	*p = 0.27*	*p = 0.718*
**GR**	L-PRF+ABG	1 ± 0.57 (0.8 to 1.2)	1.66 ± 0.48 (1.48 to 1.83)	0.66 ± 0.48 (0.48 to 0.83)	0.59 ± 0.45 (0.43 to 0.75)
	CM+ABG	1.22 ± 0.79 (0.93 to 1.5)	1.97 ± 0.54 (1.77 to 2.16)	0.78 ± 0.71 (0.53 to 1.04)	0.85 ± 0.45 (0.69 to 1.01)
	*diff*	*p = 0.21*	*p = 0.017*	*p = 0.41*	*p = 0.029*
**DBL**	L-PRF+ABG	9.62 ± 1.04 (9.25 to 10)	6.69 ± 0.97 (6.34 to 7.04)	2.94 ± 0.76 (2.66 to 3.21)	3.04 ± 0.68 (2.8 to 3.29)
	CM+ABG	10.25 ± 1.57 (9.69 to 10.81)	7.53 ± 1.22 (7.09 to 7.97)	2.72 ± 0.85 (2.41 to 3.03)	2.61 ± 0.68 (2.37 to 2.85)
	*diff*	*p = 0.65*	*p = 0.003*	*p = 0.28*	*p = 0.015*

PPD: pocket probing depth. CAL: clinical attachment level. GR: gingival recession. DBL: defect bone level. SD: standard deviation of the outcome. CM+ABG: defects treated by Collagen membrane + inorganic bovine bone combination. L-PRF+ABG: Defects treated by L-PRF + inorganic bovine bone combination. N.B. All *p*-values in observed scores columns refer to two-tail ANOVAs analyses (based on observed means). The Baseline–12 months follow-up estimated marginal column reports two-tail ANCOVA analyses, with SDs calculated from SEs. No *p*-value was adjusted for multiplicity.

**Table 2:** Differences between treatments in clinical and radiographic parameter changes in mm (Means ± SE) from Baseline to 12 Months with 90% Confidence Intervals.

Parameter	Treatment	ANCOVA	ANOVA
		Estimated Mean ± SE (90% CI)	Observed Mean ± SE 90% CI
**CAL Gain**	L-PRF+ABG	3.38 ± 0.118 (3.18 to 3.57)	3.44 ± 0.198 (3.11 to 3.77)
	CM+ABG	3.32 ± 0.134 (3.10 to 3.54)	3.75 ± 0.198 (3.42 to 4.08)
	**Mean Difference**	**Estimated Mean ± SE** **(90% CI)** **(*Simultaneous Bonferroni-Nyholt 90% CI*)**	
**CAL Gain**	CM+ABG–L-PRF+ABG	−0.0564 ± 0.155 (−0.316 to 0.203) (*−0.399 to 0.287*)	0.312 ± 0.28 (−0.155 to 0.789)
**PPD Reduction**	CM+ABG–L-PRF+ABG	0.232 ± 0.13 (0.015 to 0.449) (*−0.055 to 0.519*)	0.219 ± 0.325 (−0.323 to 0.761)
**DBL Gain**	CM+ABG–L-PRF+ABG	−0.433 ± 0.173 (−0.721 to −0.145) (*−0.814 to −0.052*)	−0.219 ± 0.202 (−0.555 to 0.118)
**GR Increase**	CM+ABG–L-PRF+ABG	0.255 ± 0.114 (0.0645 to 0.445) (*0.003 to 0.506*)	0.125 ± 0.151 (−0.128 to 0.378)

PPD: pocket probing depth. CAL: clinical attachment level. GR: gingival recession. DBL: defect bone level. SE: standard error of the mean. CM+ABG: defects treated by collagen membrane + autologus bone graft. L-PRF+ABG: Defects treated by L-PRF + autologus bone graft. NB. The estimated marginal means are evaluated at the following average values of covariates: CAL_t0_ = 8.468. PPD_t0_ = 7.453. GR_t0_ = 1.109. DBL_t0_ = 9.937.

## Data Availability

The data is available upon reader request.
